# Investigation of Warpage for Multi-Die Fan-Out Wafer-Level Packaging Process

**DOI:** 10.3390/ma15051683

**Published:** 2022-02-23

**Authors:** Chuan Chen, Meiying Su, Rui Ma, Yunyan Zhou, Jun Li, Liqiang Cao

**Affiliations:** Institute of Microelectronics, Chinese Academy of Sciences, Beijing 100029, China; chenchuan@ime.ac.cn (C.C.); sumeiying@ime.ac.cn (M.S.); marui@ime.ac.cn (R.M.); lijun@ime.ac.cn (J.L.)

**Keywords:** fan-out wafer-level packaging, viscoelastic, warpage, multi-die

## Abstract

This paper focuses on characterizing the evolution of warpage, effects of epoxy molding compound (EMC), and effects of carrier 2 (the second carrier in the process) of 12 inch RDL-first multi-die fan-out wafer-level packaging (FOWLP) during the manufacturing process. The linear viscoelasticity properties of EMC and polyimide (PI) were characterized using dynamic mechanical analysis (DMA) in the frequency domain at different temperatures., The elastic and viscoelastic model were used for PI and EMC, the finite element analyses (FEA) of the cured structure were carried out and the results were compared with the test results. The viscoelastic properties of the EMC in the FEA could predict the wafer warpage more accurately. The FEA and experiments were used to investigate the evolution of warpage. The molding had a great influence on the warpage. The effects of the EMC and carrier 2 were also investigated with FEA. The wafer warpage could be reduced by lowering the thickness of the EMC, increasing the thickness of carrier 2, and selecting EMC and carrier 2 with a matched coefficient of thermal expansion (CTE).

## 1. Introduction

In recent years, consumer electronics, such as smart mobile devices, 5G, artificial intelligence, etc., have witnessed explosive growth. Consumers have increasingly higher requirements for electronic products, such as small size, low cost, high performance, and low power consumption. As Moore’s law is slowing down, the international technology roadmap for semiconductors (ITRS) has defined the two major technical routes for parallel development: more Moore and more than Moore [1]. More Moore refers to the use of new manufacturing techniques to promote Moore’s Law. More than Moore refers to the optimization of integrated circuits by relying on new circuit designs and system algorithms. Heterogeneous and homologous integration through the system in package (SiP) is one of the most important technologies for more than Moore due to its low cost and appropriate technical challenges. It can meet the requirements of consumers for electronic products, so it has been widely investigated [2,3].

Currently, advanced technologies to implement SiP include 2.5D [4] and 3D [5] packaging, but their cost, reliability, thermal management, and assembly are all facing great challenges. The fan-out wafer-level packaging (FOWLP) is a very good solution that can satisfy the needs for greater feasibility and enabling SiP. To overcome the problem wherein the number of pins is limited by the chip area, Infineon Technologies first developed the FOWLP known as embedded wafer-level ball grid array (eWLB) [6]. Except for eWLB, some other types of fan-out packaging, such as panel-level fan-out packaging [7], silicon wafer integrating fan-out technology [8], and integrated fan-out technology [9], have been developed. According to the different embedded materials, FOWLP can be divided into three types: embedded substrate technology, embedded molding compound technology, and embedded other structure technology. The FOWLP embedded in molding compounds can be divided into molding first and redistribution layer (RDL) first [10]. As chips are embedded in other materials, such as molding compounds, warpage is inevitable during the process due to cured-induced shrinkage and mismatch of the coefficient of thermal expansion (CTE) among materials and chips [11,12]. Warpage is a critical issue to subsequent processes because of misalignment and difficulty in handling, such as RDL, ball placement, and wafer singulation. Moreover, serious warpage will degrade the reliability and even lead to serious failures such as delamination and breakage. For the large-sized wafer-level process and panel-level process, the warpage is particularly serious. Several studies have been conducted on the research and control of the warpage of FOWLP.

Deng et al. [13] studied the effects of the thermal and chemical shrinkage of EMC on the warpage of FOWLP after the curing process through theoretical and experimental analysis. The viscoelastic property of the EMC was not considered. Liu et al. [14] used the approaches of CTE balance to adjust the warpage of the panel. Yeh et al. [15] studied the warpage of a reconstructed molded wafer during the curing process with FEA. The results showed that the curing process conditions have a great influence on the warpage. The viscoelastic property of the molding material was considered. Takekoshi et al. [16] studied the effects of composing a material combination of an RDL-first-type FOWLP with various die occupancy ratios during the fabrication processes on warpage through both experiments and numerical simulation. The results showed that the warpage could be controlled within 1 mm during all the fabrication process steps. Lau et al. [17] studied the warpage of a chip-first FOWLP during the processes with experiments and FEA. The FOWLP had three redistributed layers with a very large die. Cheng et al. [18] characterized the warpage evolution of FOWLP during the wafer-level molding process with a simulation methodology. The effects of thermal annealing and wafer thinning on the warpage were explored. The viscoelastic property of the EMC was characterized using DMA and was considered in FEA. Chen et al. [19] developed a wafer-level warpage model for the 12 inch mold-first-type FOWLP with the model change technique. The predicted warpage results showed a good correlation with the experimental results. The viscoelastic properties of EMC and dielectrics were considered in the model and found to be critical for warpage prediction. In the above studies, the effects of EMC properties, structural dimensions, and processes on warpage under a single die were studied. Except for reference [19], other studies only consider the viscoelastic property of EMC.

In this paper, the multi-die FOWLP structure and process are introduced. Then, the viscoelastic properties of EMC and PI are measured. The simulation warpage using viscoelastic and elastic properties, respectively, for a 12 inch RDL-first FOWLP is compared with the measured warpage to evaluate the accuracy of the viscoelastic properties. Finally, the evolution of warpage is investigated with FEA and experiments, and the effects of EMC and carrier 2 are investigated with FEA.

## 2. Structure and Manufacturing Process of the FOWLP

### 2.1. Structure

**Figure 1:**
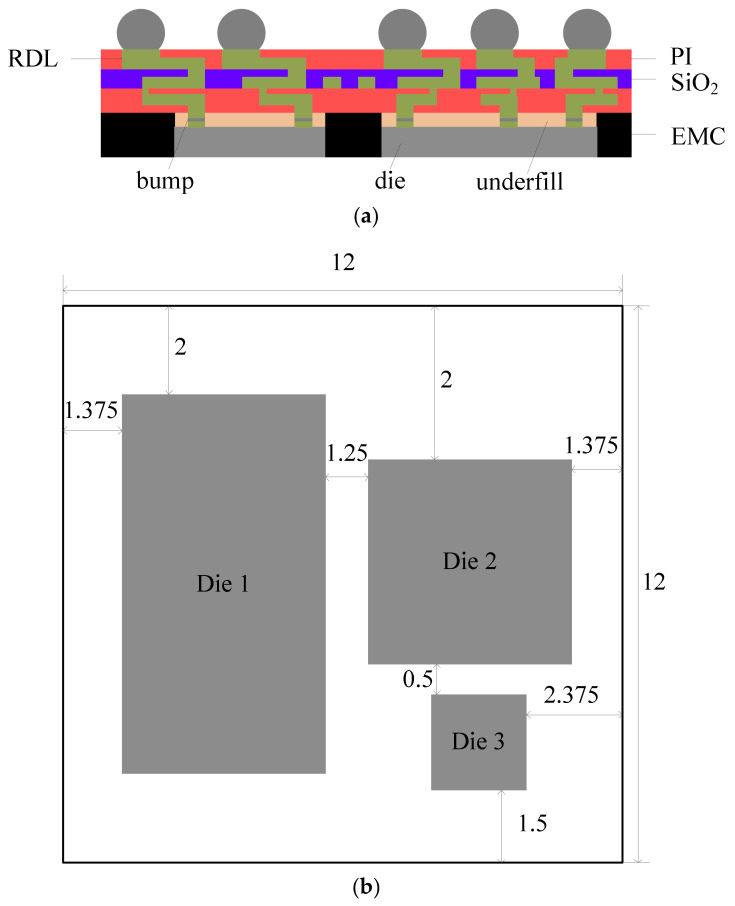
(**a**) The structure of the FOWLP; (**b**) top view of the FOWLP (unit: mm).

The fan-out packaging studied in this paper contains three dies, three layers of RDL, and three layers of PI, as shown in Figure 1. The die size is shown in Table 1 and the package size is 12 mm× 12 mm. The thickness of the interconnection is shown in Table 2.

### 2.2. Process

**Figure 2:**
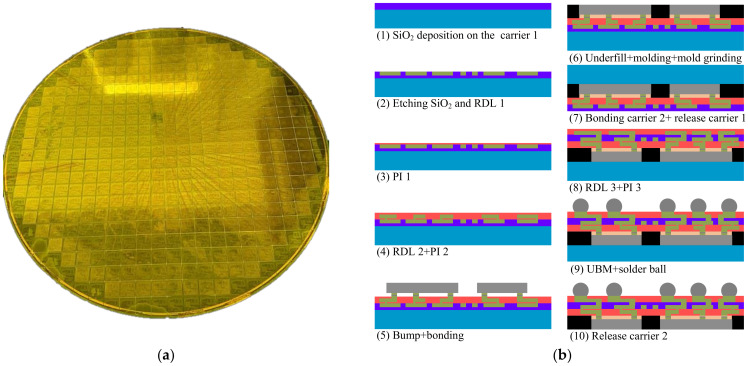
(**a**) The optical photo of FOWLP; (**b**) the manufacturing process of the FOWLP.

In this paper, the FOWLP was manufactured using a 12 inch wafer with the RDL-first process. To control the warpage of the wafer during the process, the carrier was used twice, and was named carrier 1 and carrier 2, respectively. The FOWLP manufacturing process is shown in Figure 2b. (1) A layer of 3 μm SiO_2_ was deposited on the silicon wafer (carrier 1). (2) SiO_2_ was etched by dry etching to form a route groove and 2 μm RDL 1 was manufactured in SiO_2_. Then, (3) and (4), 5 μm PI 1, 4 μm RDL 2, and 7 μm PI 2 were made. (5) Bumps were made and dies were bonded with bumps. (6) The bottom of each die was filled with underfill and EMC was molded with a thickness of 500 μm; then, the EMC was ground to 250 μm. (7) Carrier 2 was bonded and carrier 1 was released with a silicon-less process. The material of carrier 2 could be silicon or glass. (8) Then, 5 μm RDL 3 and 8 μm PI 3 were made. (9) UBM was made and solder paste was printed to form balls with reflow. (10) Carrier 2 was released. The interconnect line and bump were copper in the above process.

## 3. Material Properties Characterization

### 3.1. Linear Viscoelasticity

In FEA, EMC is often simulated as an elastic material. However, as a polymer, EMC usually has viscoelastic properties, meaning that its modulus is not only related to temperature but also related to loading, such as the strain rate. Viscoelasticity obeys the time–temperature superposition (TTS) principle. According to TTS, the curves of modulus at different temperatures can be translated to generate the master curve at a specific temperature to predict the modulus of materials beyond the measured frequency. The temperature shift factor is defined as follows:

(1) $$\alpha_{T}=\frac{f_{T}}{f_{T_{\mathrm{ref}}}},$$

where the *f_T_* is the frequency after translation according to TTS at temperature T; *f_T_ref__* is the frequency at reference temperature *T_ref_*. The shifting factor of the polymer can usually be fitted into the Williams–Landel–Ferry (WLF) equation as follows:

(2) $$\log\alpha_{T}=\frac{-C_{1}(T-T_{\mathrm{ref}})}{C_{2}+T-T_{\mathrm{ref}}},$$

where *T* is the temperature, and *T_ref_* is the reference temperature. *C*_1_ and *C*_2_ are constants for a particular polymer.

The mechanical behavior of linear viscoelasticity can be described by the following constitutive equation:

(3) $$\sigma \left(t\right)=\int_{-\infty }^{t}E\left(t-\tau \right)\frac{de}{dt}d,$$

where *σ* is the stress; *e* is the strain; *E*(*t*) is the modulus.

In the literature, the generalized Maxwell model is a linear viscoelastic model with high accuracy and wide application. The modulus *E*(*t*) is usually expressed by the Prony series form of order *n*, as shown in Equation (4), and it is expressed as Equation (5) in the frequency domain. *E’* and *E”* are the storage modulus and loss modulus, respectively, which are expressed as Equations (6) and (7) [20].

(4) $$E(t)=E_{\infty }+\sum_{i=1}^{n}E_{i}\exp\left(-t/\tau_{i}\right),$$

(5) $$E*\left(j\omega \right)=E’\left(\omega \right)+jE"\left(\omega \right),$$

(6) $$E’(\omega )=E_{\infty }+\sum_{i=1}^{n}E_{i}\frac{{\left(\omega \tau_{i}\right)}^{2}}{1+{\left(\omega \tau_{i}\right)}^{2}},$$

(7) $$E’’(\omega )=\sum_{i=1}^{n}E_{i}\frac{\omega \tau_{i}}{1+{\left(\omega \tau_{i}\right)}^{2}},$$

where *τ_i_* is the relaxation times, *e_i_* is the Prony coefficients, *ω* is the angular frequency (2π*f*), and *E_∞_* is the long-term (tensile) modulus.

The storage modulus and loss modulus at different temperatures and frequencies are measured and then translated according to TTS. By fitting the translated modulus, the characterization of the storage modulus and loss modulus in the form of Equations (6) and (7) is obtained.

### 3.2. Property Characterization

The EMC for fan-out packaging was cured on a 12 inch silicon wafer, and the conditions of curing are shown in Table 3. Then, the test samples with dimensions of 35 mm × 9.9 mm × 0.5 mm were made. The DMA system Q800 (TA Instruments, New Castle, DE, USA) was used to measure the viscoelasticity of the cured EMC in a single cantilever clamp with multi-frequency–strain mode. The temperature was 25–200 °C, the heating rate was 5 °C/min, the initial preloading amplitude was 20 μm, and the frequencies were 1, 3, 6, 10, 30 Hz.

**Figure 3:**
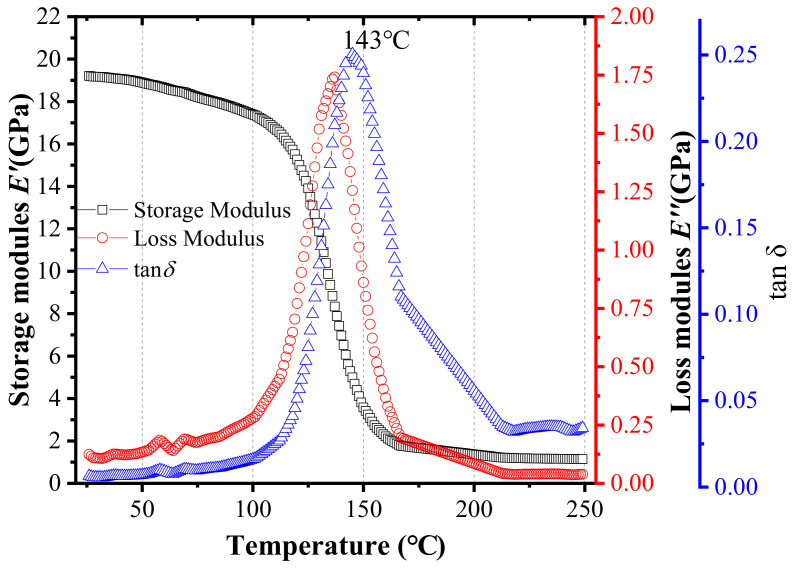
Storage modulus, loss modulus, and tan *δ* of EMC tested by DMA at 1 Hz.

Figure 3 shows the measurement results of DMA at 1 Hz. The storage modulus decreases with the increase in temperature, and the loss modulus increases first and then decreases. The ratio of loss modulus to storage modulus is defined as the loss tangent (tan *δ*). The peak of tan *δ* results from glass relaxation occurring in the EMC, and the glass transition temperature (*Tg*) is defined as the temperature corresponding to the maximum in tan *δ*. Therefore, the *Tg* of the EMC is 143 °C, as shown in Figure 3.

**Figure 4:**
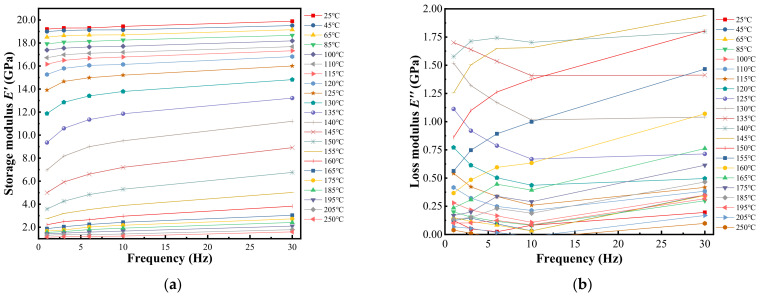
Modulus at different frequencies and isothermal temperatures. (**a**) Storage modulus; (**b**) loss modulus.

**Figure 5:**
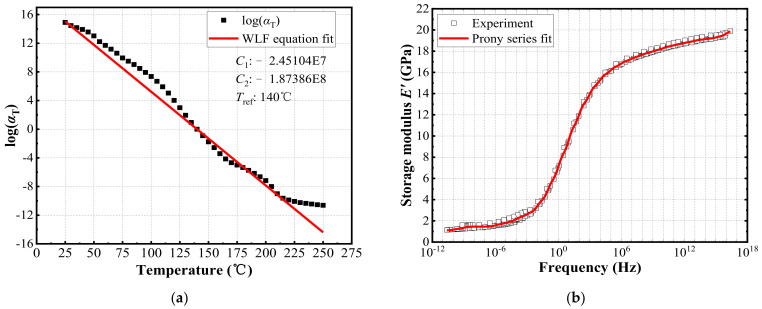
(**a**) Values of translation factor *α_T_* and WLF fitting; (**b**) Prony series fitting of the storage modulus master curve at 140 °C.

Figure 4 shows the storage modulus and loss modulus of EMC at different frequencies and temperatures, as measured by DMA. At the reference temperature of 140 °C, the translation factor is achieved by translating the curves at different temperatures according to TTS and Equation (1). The parameters *C*_1_ and *C*_2_ in the WLF equation are achieved by fitting the translation factor, as shown in Figure 5a. The master curve generated by translating the curves in Figure 4 was fitted into a generalized Maxwell model, as shown in Figure 5b. The fitting Prony series coefficients *E_i_* and *τ_i_* of EMC are listed in Table 4. The relative modulus *α_i_* is required in Ansys and was calculated as follows:

(8) $$\alpha_{i}=E_{i}/E_{0}$$

(9) $$E_{0}=E_{\infty }+\sum_{i=1}^{n}E_{i},$$

As a polymer, PI can be characterized by viscoelastic properties. In this study, a PI for wafer-level packaging was measured. The curing conditions for the 12 inch wafer are shown in Table 5. The size of the sample was 30 mm × 9.2 mm × 0.01 mm. Because the sample was a film, the film tensile fixture was used. The frequencies were 3, 6, 10, 30 Hz, the temperature was 25–260 °C, the heating rate was 3 °C/min, and the initial preloading amplitude was 20 μm. At the reference temperature of 195 °C, the translation factor is achieved by translating the curves at different temperatures according to TTS and Equation (1). The parameters *C*_1_ and *C*_2_ in the WLF equation are achieved by fitting the translation factor, as follows:

(10) $$\log\alpha_{T}=\frac{1.23765e^{15}(T-195)}{-1.75325e^{16}+T-195},$$

**Figure 6:**
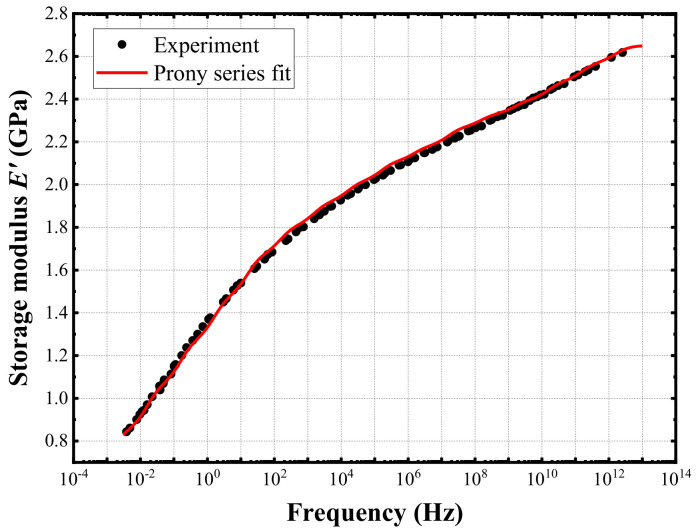
Prony series fitting of the storage modulus master curve at 195 °C.

The master curve generated by translating the curves at different temperatures was fitted into a generalized Maxwell model, as shown in Figure 6. The fitting Prony series coefficients of EMC are listed in Table 6.

### 3.3. Property Verification

**Figure 7:**
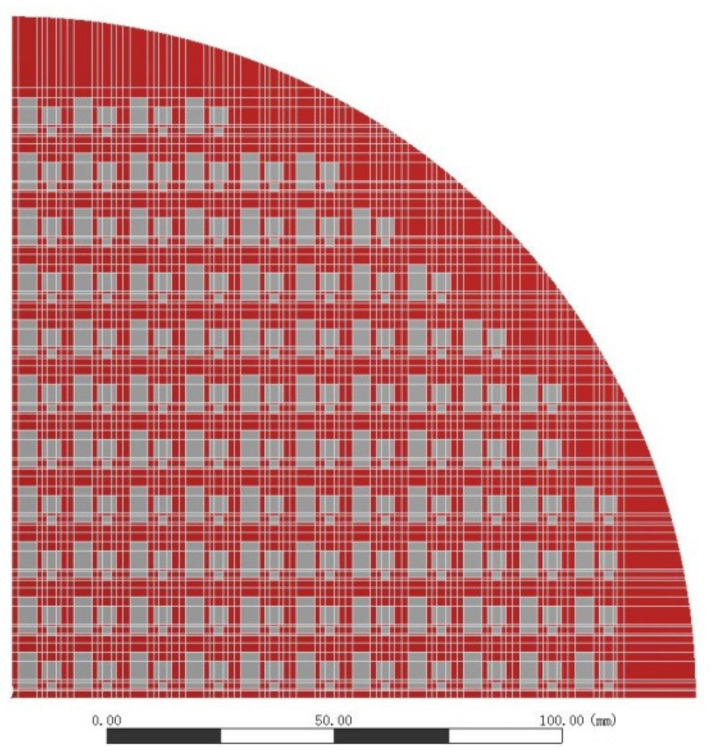
The 1/4 shell element model of the wafer.

In order to verify the accuracy of the PI and EMC viscoelastic models, the finite element analysis of the cured structure was carried out by using the PI and EMC elastic model and viscoelastic model, respectively, and the results were compared with the test results. In this study, the shell element was used. To improve the efficiency, a 1/4 model was established, as shown in Figure 7. The elastic properties of materials used in FOWLP are shown in Table 7. Carrier 1 was a silicon wafer and the thickness was 0.775 mm.

**Figure 8:**
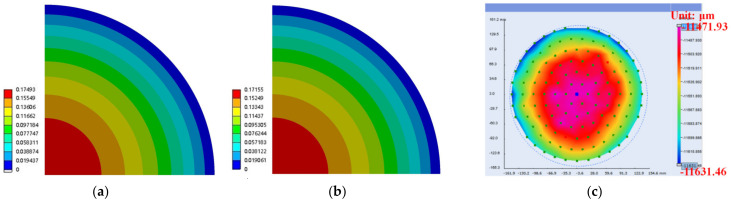
The warpage contour of 1/4 model after PI 2 curing and cooling down to room temperature. (**a**) Simulation with elasticity; (**b**) simulation with viscoelasticity; (**c**) measurement.

In the process, PI was cured under the conditions shown in Table 5 and then cooled to 25 °C for 3 h. The warpage of the wafer was simulated after PI 2 curing, and the effect of RDL in silicon and PI 1 was not considered. The reference temperature (the temperature at zero stress) was set as 120 °C, which is the curing temperature of PI. The simulation warpage (carrier 1 is at top and crying face is positive) of PI using the elastic model and viscoelastic model described in this paper was 0.175 mm and 0.172 mm, respectively, as shown in Figure 8a,b. The T-MAP measuring instrument of the Fogale Nanotech Company was used for the experimental measurement. The top side was carrier 1, and the warpage was 0.160 mm, as shown in Figure 8c. Therefore, the warpage considering the viscoelastic properties of the polymer is very close to that using elastic properties.

**Figure 9:**
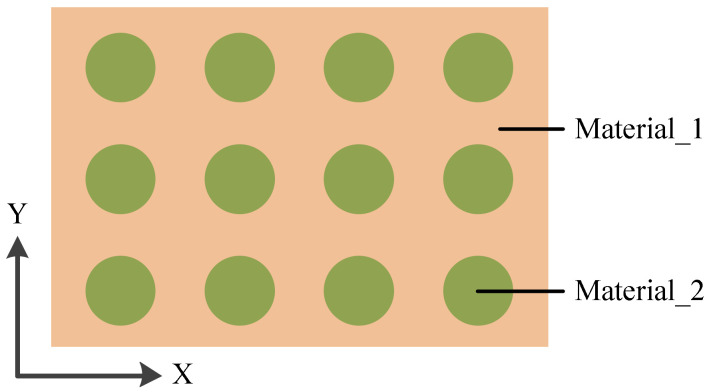
The model of the bi-material composite structure.

In the process, EMC was cured under the conditions shown in Table 3 and then cooled to 25 °C for 3 h. The warpage of the wafer was simulated after EMC curing, and the effects of SiO_2_ and RDL were ignored. The reference temperature was set as 125 °C, which is the curing temperature of EMC. To improve the computational efficiency, the equivalent formulas of the composite material were used to calculate the mechanical properties of the underfill and bump layers. Figure 9 shows a typical bi-material composite structure, and its mechanical properties can be described by Equations (11)–(18).

(11) $$E_{x}=E_{y}=E_{1}E_{2}/(V_{2}E_{1}+V_{1}E_{2}),$$

(12) $$E_{z}=V_{1}E_{1}+V_{2}E_{2},$$

(13) $$\mu_{\mathrm{xz}}=\mu_{\mathrm{yz}}=E_{x}(V_{1}\mu_{1}+V_{2}\mu_{2})/E_{z},$$

(14) $$\mu_{\mathrm{xy}}=V_{2}\mu_{2}+V_{1}\mu_{1}\frac{\left[1+\mu_{1}-\left(\frac{E_{1}}{E_{z}}\right)\mu_{\mathrm{xz}}\right]}{\left[1-\mu_{1}^{2}+\mu_{1}\left(\frac{E_{1}}{E_{z}}\mu_{\mathrm{xz}}\right)\right]},$$

(15) $$\alpha_{z}=\left[V_{1}\alpha_{1}E_{1}+V_{2}\alpha_{2}E_{2}\right]/E_{z},$$

(16) $$\alpha_{y}=\alpha_{1}V_{1}\left(1+\mu_{1}\right)+\alpha_{2}V_{2}\left(1+\mu_{2}\right)-\alpha_{z}\mu_{\mathrm{xz}},$$

$$\alpha_{x}=\alpha_{y},$$

(17) $$G_{\mathrm{xy}}=E_{x}/\left[2\times \left(1+\mu_{\mathrm{xy}}\right)\right],$$

(18) $$G_{\mathrm{xz}}=G_{\mathrm{yz}}=G_{1}G_{2}/\left(V_{2}G_{1}+V_{1}G_{2}\right),$$

where *V*, *E*, *G*, *α*, and *μ* are the volume percentages, Young’s modulus, shear modulus, CTE, and Poisson’s ratio of the material, respectively. The subscript “1” represents material_1 and the subscript “2” represents material_2. The subscripts “x”, “y”, and “z” represent the X direction, Y direction, and Z direction, respectively. The subscript “xy” represents in-plane, and the subscripts “xz” and “yz” represent vertical.

The equivalent mechanical properties of the underfill and bump layers of three dies were calculated, which are shown in Table 8.

**Figure 10:**
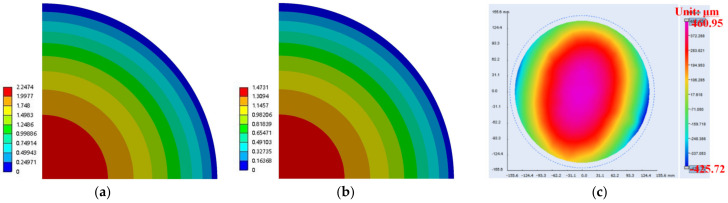
The warpage contour of 1/4 model after EMC curing and cooling down to room temperature. (**a**) Simulation with elasticity; (**b**) simulation with viscoelasticity; (**c**) measurement.

The warpage of the wafer (carrier 1 is at top and crying face is positive) is shown in Figure 10 after molding and cooling down to 25 °C. The warpage of the elastic model is 2.247 mm, that of the viscoelastic model is 1.473 mm, and the measurement result is 0.887 mm. The error between simulation and measurement is large, but the results show that the warpage of the viscoelastic model is closer to the measurement values. Therefore, the EMC viscoelastic model described in this paper is able to accurately predict the warpage.

## 4. Results and Discussion

### 4.1. Evolution of Warpage

**Figure 11:**
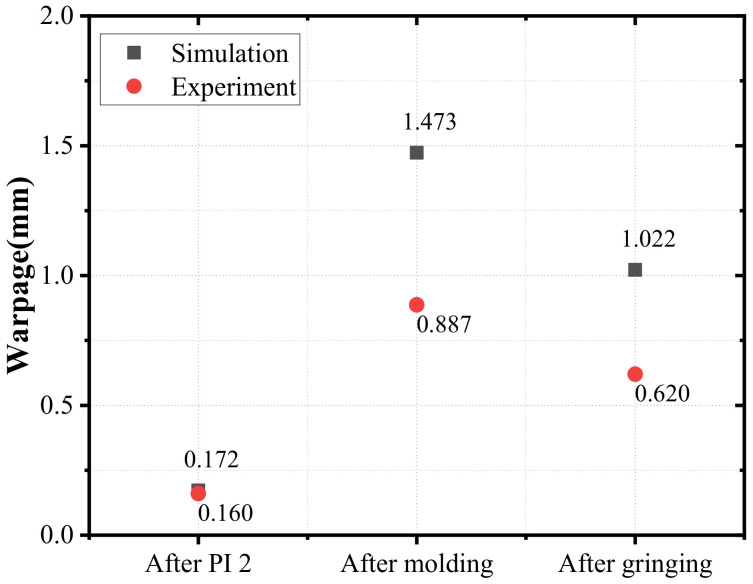
The evolution of warpage in the process (carrier 1 is at top and crying face is positive).

FEA was used to simulate the warpage after PI 2 curing, molding, and mold grinding in order to study the evolution of the wafer warpage in the process. The results of FEA are 0.172 mm, 1.473 mm, and 1.022 mm, respectively, as shown in Figure 11. Correspondingly, the warpage is 0.16 mm, 0.887 mm, and 0.62 mm in the experiment. It can be seen that the wafer warpage increases significantly after the molding, which indicates that the molding has a great influence on the warpage. This is due to the very large difference in the thermal expansion coefficient between EMC and other materials. Therefore, it is necessary to research the molding process. The difference between simulation and experimental values is remarkable. According to the conclusion made in Section 3.3, compared with linear elasticity, the simulation based on viscoelasticity is closer to the measured value. However, the prediction accuracy of warpage is affected by other factors, such as volume shrinkage during cure, besides the selection of the elastic model.

### 4.2. Effects of EMC

**Figure 12:**
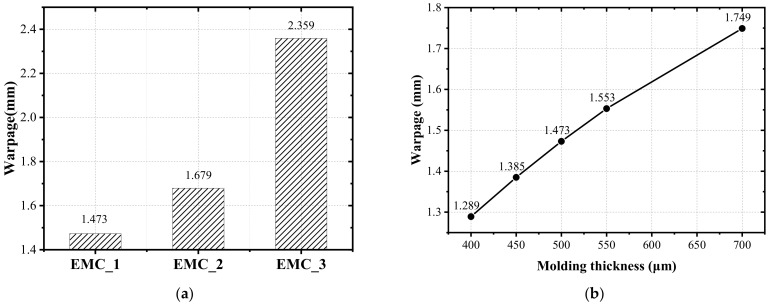
(**a**) The effect of different EMCs on wafer warpage; (**b**) The effect of thickness of EMC on wafer warpage (carrier 1 is at top and the crying face is positive).

The change in the EMC properties will lead to different warpage. Therefore, FEA was used to simulate the effects of the other two EMCs (EMC_2 and EMC_3) on the warpage. The linear elastic properties of the two materials are shown in Table 9. With the exception that CTE is different from EMC_1, all other parameters are the same. Figure 12a shows the simulation warpage after molding. It can be seen that the warpage molding with EMC_2 is 1.679 mm, which is larger than molding with EMC_1. The warpage molding with EMC_3 is the maximum, which is 2.359 mm. Therefore, the CTE of EMC has a very significant effect on warpage.

The change in the EMC thickness will also lead to different warpage. The simulation results are shown in Figure 12b. It can be seen that the warpage increases with the increase in the EMC thickness, and the increase rate is decreasing. Therefore, under the condition of process capacity, the wafer warpage can be reduced by lowering the thickness of the EMC.

### 4.3. Effects of Carrier

**Figure 13:**
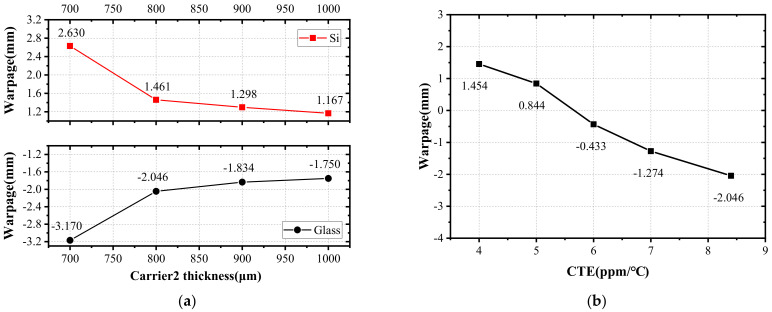
(**a**) The effect of material and thickness of carrier 2 on wafer warpage; (**b**) The effect of CTE of glass carrier 2 on warpage (carrier 2 is at top and crying face is positive).

The warpage will be varied when adjusting the material and thickness of the carrier, and the matching with silicon, EMC, and PI material. Therefore, FEA was used to simulate the effects of carrier 2 after releasing carrier 1 in step (7) in the process. The carrier 2 was bonded with the fan-out wafer at 180 °C and then cooled to 25 °C for 10 min. The material of carrier 2 was silicon and glass, respectively, and the thickness is 700 μm, 800 μm, 900 μm, and 1000 μm, respectively. The glass carrier 2 has a CTE of 8.4 ppm/°C, Young’s modulus of 71.5 GPa, and Poisson’s ratio of 0.22. The results are shown in Figure 13a, where carrier 2 is at the top and the crying face is positive. The warpage with silicon carrier 2 is lower than that with glass. With the increase in the thickness, the warpage decreases, and the reduction rate is also decreasing. Therefore, the warpage can be optimized by selecting the appropriate material and thickness of carrier 2.

Glass can provide a wide range of CTEs to match different fan-out packaging materials, and has become the preferred choice in the process of FOWLP. The effects of the CTE of glass on wafer warpage were simulated and analyzed by FEA. The trend of 12 inch FOWLP warpage when increasing the CTE of glass carrier 2 is shown in Figure 13b. It can be seen that the warpage decreases significantly with the increase in the CTE of glass. When the CTE increases from 4 ppm/°C to 8.4 ppm/°C, the warpage decreases by 3.5 mm. When the CTE is less than 5 ppm/°C, the warpage is positive, and the increase rate decreases with the decrease in CTE. When the CTE is not less than 6 ppm/°C, the warpage is negative, and the reduction rate decreases with the increase in CTE. Therefore, the warpage can be optimized by selecting a carrier 2 material with a matched CTE.

## 5. Conclusions

In this study, we measured the linear viscoelasticity properties of EMC and PI using dynamic mechanical analysis. Then, the simulation warpage of viscoelastic and elastic properties for 12 inch RDL-first FOWLP were compared with the measured warpage to evaluate the accuracy of the viscoelastic properties. Moreover, the evolution of warpage was investigated with simulations and experiments, and the effects of EMC and effects of carrier 2 were investigated with FEA. Some of the most important results are summarized below:

1. The viscoelastic properties of EMC in FEA can predict the wafer warpage more accurately than elastic properties under the process. The warpage considering the viscoelastic properties of PI is very close to that using elastic properties.
2. In the process, the molding has a great influence on the warpage. The wafer warpage can be reduced by lowering the thickness of EMC and selecting an EMC with a smaller CTE.
3. Carrier 2 also has a very significant effect on warpage. The wafer warpage can be optimized by increasing the thickness of carrier 2 and selecting a carrier 2 with a matched CTE.

## Figures and Tables

**Table 1:** The die size in the FOWLP.

Num.	Size (mm)	Thickness (μm)	Diameter of Bump (μm)	Height of Bump (μm)
Die 1	4 × 8	250	150	75
Die 2	4 × 4	350	20	25
Die 3	2 × 2	200	60	47

**Table 2:** The thickness of the interconnection in the FOWLP.

Layer	Thickness (μm)	Layer	Thickness (μm)	Layer	Thickness (μm)
PI 1	5	RDL 1	2	SiO_2_	3
PI 2	7	RDL 2	4		
PI 3	8	RDL 3	5		

**Table 3:** Process conditions for preparing cured polyimide.

Conditions of Curing				Thickness (μm)
Prepreg Curing		Post Curing		
*T* (°C)	*t* (min)	*T* (°C)	*t* (min)	
125	10	150	60	500

**Table 4:** Prony series coefficients of EMC.

*i*	*E_i_* (GPa)	*α_i_*	*τ_i_* (s)	*i*	*E_i_* (GPa)	*α_i_*	*τ_i_* (s)	*i*	*E_i_* (GPa)	*α_i_*	*τ_i_* (s)
1	0.14485	0.00727	10^10^	10	2.63466	0.13223	10^−1^	19	0.33030	0.01658	10^−10^
2	0.20816	0.01045	10^9^	11	2.19706	0.11027	10^−2^	20	0.21223	0.01065	10^−11^
3	0.23859	0.01197	10^6^	12	1.59056	0.07983	10^−3^	21	0.20900	0.01049	10^−12^
4	0.23046	0.01157	10^5^	13	1.08914	0.05466	10^−4^	22	0.21489	0.01078	10^−13^
5	0.46783	0.02348	10^4^	14	0.71367	0.03582	10^−5^	23	0.09099	0.00457	10^−14^
6	0.42749	0.02145	10^3^	15	0.52184	0.02619	10^−6^	24	0.20694	0.01039	10^−15^
7	1.11779	0.05610	10^2^	16	0.39181	0.01966	10^−7^	25	0.55236	0.02772	10^−16^
8	1.91552	0.09614	10	17	0.35507	0.01782	10^−8^	E_∞_	1.1		
9	2.49683	0.12531	1	18	0.27711	0.01391	10^−9^				

**Table 5:** Process conditions for preparing cured polyimide.

Conditions of Curing						Thickness (μm)
Prepreg Curing				Post Curing		
*T* (°C)	*t* (min)	*T* (°C)	*t* (min)	*T* (°C)	*t* (min)	
100	5	120	5	200	60	10

**Table 6:** Prony series coefficients of polyimide.

*i*	*E_i_* (MPa)	*α_i_*	*τ_i_* (s)	*i*	*E_i_* (MPa)	*α_i_*	*τ_i_* (s)
1	155.96	0.05883	100	10	70.66	0.02665	10^−7^
2	215.04	0.08112	10	11	89.02	0.03358	10^−8^
3	206.44	0.07787	1	12	60.97	0.02300	10^−9^
4	205.31	0.07745	10^−1^	13	65.46	0.02469	10^−10^
5	197.89	0.07465	10^−2^	14	87.40	0.03297	10^−11^
6	131.96	0.04978	10^−3^	15	87.26	0.03292	10^−12^
7	109.97	0.04148	10^−4^	16	77.26	0.02914	10^−13^
8	96.73	0.03649	10^−5^	E_∞_	700		
9	93.61	0.03531	10^−6^				

**Table 7:** Material properties of the FOWLP.

Material	CTE (ppm/°C)	Young’s Modulus (GPa)	Poisson’s Ratio
Si	2.8	131	0.28
Copper	17	125	0.34
SiO_2_	0.5	73	0.3
Underfill	42	7.1	0.3
PI	54	2.5	0.3
EMC	7(<Tg)/22(>Tg)	19	0.26

**Table 8:** The equivalent mechanical properties of underfill and bump layers.

	Die 1	Die 2	Die 3
CTE (ppm/°C)	X,Y 45.109 Z 33.722	X,Y 45.220 Z 32.123	X,Y 45.206 Z 31.631
Young’s modulus (GPa)	X,Y 7.683 Z 10.892	X,Y 7.897 Z 12.145	X,Y 7.976 Z 12.591
Shear modulus (GPa)	XY 2.814 XZ,YZ 2.953	XY 2.874 XZ,YZ 3.035	XY 2.898 XZ,YZ 3.065
Poisson’s ratio	XY 0.365 XZ,YZ 0.215	XY 0.373 XZ,YZ 0.200	XY 0.376 XZ,YZ 0.195

**Table 9:** The material properties of EMC_2 and EMC_3.

Material	CTE (ppm/°C)	Young’s Modulus (Gpa)	Poisson’s Ratio
**EMC_2**	8(<Tg)/30(>Tg)	19	0.26
**EMC_3**	10(<Tg)/40(>Tg)	19	0.26^1^

## Data Availability

The data presented in this study are available on request from the corresponding author. The data are not publicly available due to privacy.
